# Microwave-Assisted Rapid Green Synthesis of Gold Nanoparticles Using Seed Extract of *Trachyspermum ammi*: ROS Mediated Biofilm Inhibition and Anticancer Activity

**DOI:** 10.3390/biom11020197

**Published:** 2021-01-30

**Authors:** Kahkashan Perveen, Fohad Mabood Husain, Faizan Abul Qais, Altaf Khan, Suhail Razak, Tayyaba Afsar, Pravej Alam, Ali M. Almajwal, Mahmoud M. A. Abulmeaty

**Affiliations:** 1 Department of Botany and Microbiology, College of Science, King Saud University, 2460, Riyadh 11451, Saudi Arabia; kperveen@ksu.edu.sa; 2 Department of Food Science and Nutrition, College of Food and Agriculture Sciences, King Saud University, 2460, Riyadh 11451, Saudi Arabia; 3 Department of Ag. Microbiology, Aligarh Muslim University, Aligarh 202002, India; faizanabulqais@gmail.com; 4 Central Laboratory, Department of Pharmacology and Toxicology, College of Pharmacy, King Saud University, 2460, Riyadh 11451, Saudi Arabia; altkhan@ksu.edu.sa; 5 Department of Community Health Sciences, College of Applied Medical Sciences, King Saud University, 2460, Riyadh 11451, Saudi Arabia; tayyabaafsar12@gmail.com (T.A.); aalmajwal@ksu.edu.sa (A.M.A.); mabulmeaty@ksu.edu.sa (M.M.A.A.); 6 Department of Biology, College of Science and Humanities, Prince Sattam bin Abdulaziz University, Alkharj 11942, Saudi Arabia; alamprez@gmail.com

**Keywords:** gold nanoparticles, green synthesis, *Trachyspermum ammi*, antibiofilm, anticancer, HepG2 cell lines

## Abstract

Green synthesis of metal nanoparticles using plant extracts as capping and reducing agents for the biomedical applications has received considerable attention. Moreover, emergence and spread of multidrug resistance among bacterial pathogens has become a major health concern and lookout for novel alternative effective drugs has gained momentum. In current study, we synthesized gold nanoparticles using the seed extract of *Trachyspermum ammi* (TA-AuNPs), assessed its efficacy against drug resistant biofilms of *Listeria monocytogenes* and *Serratia marcescens*, and evaluated its anticancer potential against HepG2 cancer cell lines. Microwave-assisted green synthesis of gold nanoparticles was carried out and characterization was done using UV-vis spectroscopy, X-ray diffraction (XRD), transmission electron microscopy (TEM), and dynamic light scattering (DLS). Most nanoparticles were observed as spherical and spheroidal with few anisotropies with an average crystalline size of 16.63 nm. Synthesized TA-AuNPs demonstrated significant biofilm inhibitory activity against *L. monocytogenes* (73%) as well as *S. marcescens* (81%). Exopolysaccharide (EPS), motility, and CSH, key elements that facilitate the formation and maintenance of biofilm were also inhibited significantly at the tested sub-minimum inhibitory concentrations (sub-MICs). Further, TA-AuNPs effectively obliterated preformed mature biofilms of *S. marcescens* and *L. monocytogenes* by 64% and 58%, respectively. Induction of intracellular ROS production in TA-AuNPs treated bacterial cells could be the plausible mechanism for the reduced biofilm formation in test pathogens. Administration of TA-AuNPs resulted in the arrest of cellular proliferation in a concentration-dependent manner. TA-AuNPs decrease the intracellular GSH in HepG2 cancer cell lines, cells become more prone to ROS generation, hence induce apoptosis. Thus, this work proposes a new eco-friendly and rapid approach for fabricating NPs which can be exploited for multifarious biomedical applications.

## 1. Introduction

Nanobiotechnology is one the most promising and emerging disciplines of science that emphasizes the development of new materials that can be used in healthcare and environmental settings [1,2]. The interest in nanomaterials has increased owing to their novel optoelectronic, physicochemical, and magnetic properties compared to their bulk form [3]. The novel biological, chemical, or physical properties of nanomaterials is mainly because of their size (usually less than 100 nm) that increases their surface area to volume ratios drastically. The most common methods for the synthesis of metal nanoparticles are chemical, physical, and biological methods. In last two decades, the fabrication of metal nanoparticles using biological materials have obtained considerable attention and scientists have developed numerous novel methods of the green synthesis [4,5]. The biological synthesis (commonly called as green synthesis) of nanomaterials is advantageous over other procedures owing to the use of renewable biological materials and without producing toxic byproducts [6,7]. The green synthesis of nanoparticles used biological entities such as plant materials, microbes, etc. Another advantage of biological synthesis is its ability to produce stability nanomaterials with precise dimensions at the economic cost [8]. However, there are certain limitations/disadvantages associated with the green synthesis of metallic nanoparticles. The major disadvantage is the changes in phytochemical profile of the plant extracts due to seasonal or climate variations that may affect the synthesis procedures and bioactivity. Such changes may lead to concerns regarding the reproducibility of NPs with the same characteristics [9]. Moreover, green synthesis of metals nanoparticles is usually a one-step procedure and sometimes, nanoparticles can be prepared even in a few minutes [10]. Therefore, the green synthesis of metal nanoparticles for biomedical applications has received considerable attention due to their possibility of being developed as novel material with biological applications [11,12].

In last few decades, there has been tremendous increase in the emergence and spread of drug resistance among bacterial pathogens. This antimicrobial resistance is now a global concern as it has become one of the major contributors in mortality and morbidity diseases [13]. Moreover, multiple drug-resistance also worsens the treatment of other infections associated with chronic illness such as diabetes and cancer by reducing the effectiveness of antibiotics [14,15]. Earlier it was thought that bacteria grow in planktonic state, but later it was found that most bacteria live in complex structures called biofilms. The bacterial biofilms are complex systems comprised of bacterial communities and extracellular polymeric substances (EPS). The EPS act as protective barrier from external environment [16]. In biofilms, there is altered expression of many phenotypes (mainly virulent) compared to planktonic growth. The importance of biofilms can be understood from the National Institute of Health (NIH) estimates that roughly 80% of infections are encouraged and established by biofilms [17,18]. A vast majority of infections are caused due to the development of biofilms either by pathogenic or opportunistic pathogens [19,20].

Bacteria residing in human gut and skin as normal flora are harmless and perform a number of important functions for the host [21]. However, there are certain bacteria which are pathogenic in nature. One of the common entry points of such pathogenic bacteria is through the gastrointestinal tract through which foodborne pathogens enter. Such foodborne pathogens enter usually come from contaminated food and water. Foodborne pathogens are a major cause of food poisoning and foodborne diseases and hence pose serious threat to human health and food safety [22]. The food gets contaminated by many bacteria during processing, harvesting, handling operations, and transportation [23]. The food spoilage by microbes is the major cause of spoilage that poses a human burden for food industry [24]. The major foodborne pathogens are *Acinetobacter* spp., *Bacillus cereus*, *B. subtilis*, *Citrobacter koseri*, *Campylobacter jejuni, Serratia marcescens*, *Clostridium difficile*, *E. cloacae*, *Escherichia coli*, *Klebsiella* spp., *Listeria monocytogenes*, *Staphylococcus aureus*, *Vibrio cholerae* etc. [25,26].

In this study, gold nanoparticles were synthesized using seed extract of *Trachyspermum ammi*. The nanoparticles were characterized using UV-vis spectroscopy, X-ray diffraction (XRD), transmission electron microscopy (TEM), and dynamic light scattering (DLS). The antibiofilm activity of gold nanoparticles was checked against two foodborne bacterial pathogens viz *Serratia marcescens* and *Listeria monocytogenes*. Moreover, the anticancer activity was also assessed against HepG2 cell line.

## 2. Material and Methods

### 2.1. Synthesis and Characterization of Gold Nanoparticles (AuNPs) Using T. ammi Seed Extract

For the preparation of aqueous extract of *Trachyspermum ammi* seeds, 20 g seed powder was mixed in 500 mL double distilled water. The suspension was left for extract for 24 h followed by centrifugation to settle down the debris. The extract was filtered with 0.22 µm filter and stored at −20 °C for further use. The method synthesis was adopted from an earlier report [27]. A 10 mM stock solution of HAuCl_4_ was prepared in double distilled water. For the synthesis of gold nanoparticles, 6 mL *T. ammi* ml extract was added to 2 mL HAuCl_4_ solution and placed on a magnetic stirrer for proper mixing for 30 min. The reaction mixture was then placed in microwave oven for 2 min (2.45 GHz, 300 W). Following microwave irradiation, the colour of the reaction mixture changed from light yellow color to ruby red indicating the formation of gold nanoparticles. The colloidal suspension of gold nanoparticles was centrifuged at 20,000 rpm for 30 min to pellet down the nanoparticles.

The preliminary characterization of gold nanoparticles was done by observing the UV-vis spectrum using Shimadzu UV1900. The absorption spectrum was recorded from 350 to 700 nm using double distilled as blank.

The X-ray diffraction pattern analysis of gold nanoparticles was performed using X-ray diffractometer (Phillips X’pert; MPD 3040, EA Almelo, The Netherlands). CuKα radiation (λ = 1.54430 Å) was used to record the diffraction pattern of gold nanoparticles in 2θ range of 20–80 degrees (°) [28]. The average particle size of gold nanoparticles was obtained using Debye-Scherrer’s formula.

The size of nanoparticles and their size-distribution was characterized using DynaPro-TC-04 dynamic light scattering (DLS) instrument attached to ZetaSizer (Malvern, UK). The colloidal aqueous solution of gold nanoparticles was sonicated for 20 min before DLS measurements.

For transmission electron microscopy, 10 µL colloidal solution of gold nanoparticles was placed on the grid TEM. The grid was then air-dried completely at room temperature. Finally, grid placed in transmission electron microscope for analysis. The size distribution of gold nanoparticles was obtained by measuring the size of nanoparticles. The TEM analysis was carried out using JOEL-2100 (Tokyo, Japan) at University Sophisticated Instrumentation Facility (USIF), AMU, Aligarh, India.

### 2.2. Evaluation of Minimum Inhibitory Concentration (MIC)

*Listeria monocytogenes* ATCC 19,114 and *Serratia marcescens* ATCC 13,880 were used in the present investigation to assess the antibacterial and antibiofilm activity of the synthesized AuNPs. Antibacterial potential of AuNPs was assessed in terms of MIC using the standard microbroth dilution method described previously [29].

### 2.3. Biofilm Inhibition Activity

Polystyrene microtiter plate assay using crystal violet as the staining dye was employed to determine the biofilm inhibitory potential of synthesized AuNPs at sub-MICs (0.06–0.5× MICs) against *L. monocytogenes* and *S. marcescens* [30].

### 2.4. Extraction and Quantification of Exopolysaccharides (EPS)

Effect of sub-MICs of AuNPs on EPS was determined by quantifying the total carbohydrate content of the bacterial cells as described previously [31]. The group without AuNPs treatment was considered as the control set.

### 2.5. Swarming Motility Assay

Swarming behavior of test bacteria was assayed by point inoculating the overnight grown cultures on 0.5% Luria Bertani (LB) soft agar plates amended with sub-MICs of AuNPs. Inoculated plates were incubated for 24 h and diameter of the swarm was recorded post incubation [32].

### 2.6. MATH Assay

Cell surface hydrophobicity (CSH) is the measure of the ability of the bacteria to adhere to the substratum. Therefore, CSH of treated and untreated *S. marcescens* and *L. monocytogenes* was determined using microbial adhesion to hydrocarbon (MATH) assay [33]. Briefly, bacteria were added to tubes containing 3 mL of LB broth with or without sub-inhibitory concentrations of AuNPs and left for incubation for 24 h. Hydrocarbon (toluene) was added to the tubes and vortexed vigorously for 180 s. Tubes were left undisturbed for 10 min to separate out the phases, lower aqueous phase was collected and absorbance was read at 600 nm before and after vortexing.

### 2.7. Disruption of Mature (Preformed) Biofilm

One day (24 h) old biofilms of *S. marcescens* and *L. monocytogenes* formed in 96-well microtiter plate was treated with sub-MICs of AuNPs and again incubated for 24 h duration. After incubation, growth media was removed from wells and adhered cells were washed thrice, stained with crystal violet, and absorbance was read at 585 nm to assess the disruption of preformed biofilm [34].

### 2.8. ROS Generation

Levels of ROS generated in cells of test pathogen upon treatment with AuNPs was evaluated using a fluorescent probe 2,7-dichlorofluorescein diacetate (DCHF-DA) as described previously [35].

### 2.9. Cell Viability Assay/MTT Assay

3-(4, 5-dimethylthiazol-2-yl)-2, 5-diphenyl tetrazoliumbromide protocol was performed to show the effect of TA-AuNPs on the viability of HepG2 cell lines. The cells were seeded (1 × 10^4^ cells per well) in 1 mL of culture medium consisting of 10–200 µg/mL dilution of TA-AuNPs in 24-well microtiter plates. Cells were kept in a humidified incubator for 48 h at 37 °C, 200 µL of 3–94,5-dimethylthiazol-2-yl)-2,5-diphenyl tetrazoliumbromide (5 mg/mL phosphate buffer saline, PBS) was supplemented to each well and kept for 2 h, 200 µL of DMSO was added to each plate which was then spun (1800× *g* for 5 min at 4 °C). The readings at 540 nm wavelength were noted on a microplate reader (Elx 800). Effect of TA-AuNPs on inhibition of growth was calculated as % cell viability as DMSO-administrated cells were retained as control. Absorbance numbers of media containing wells were subtracted from test sample values.

$$\%\;Cell\;viability=\;\frac{Absorbance\;of\;sample-absorbance\;of\;blank}{Absorbance\;of\;DMSO-absorbance\;of\;blank}\;\times 100$$

### 2.10. Protein Determination (Bradford Assay)

Bradford assay was employed for determination of protein content from cell pellets obtained from HepG2 cancer cells treated with TA-AuNPs [36].

### 2.11. Measurement of GSH Levels in HepG2 Cancer Cell LINES

HepG2 cancer cells treated with TA-AuNPs were centrifuged and cellular protein was precipitated. Consequently, 0.4 M, (pH−8.9) Tris buffer and DTNB were added to the supernatant consecutively and incubated for 10 min on shaking at 37 °C. Color intensity was measured at 412 nm [37].

### 2.12. Estimation of Lipid Peroxidation in HepG2 Cancer Cell Lines

TBARS assay was used for estimation of lipid peroxidation (LPO). TA-AuNPs administrated HepG2 cells were centrifuged, sonicated, and then again centrifuged. Supernatant was collected and 1 mL of thiobarbituric acid (TBA) was added to 500 µL of supernatant and incubated for 15 min at 100 °C in water bath. The reaction mixture was allowed to cool before cell centrifugation at 13,000× *g* for 2 min. A total of 500 µL of lysate supernatant was removed and fluorescent adduct was measured at 550 nm. TBARS are expressed as MDA equivalents [38].

## 3. Results and Discussion

### 3.1. Synthesis and Characterization of AuNPs

**Figure 1:**
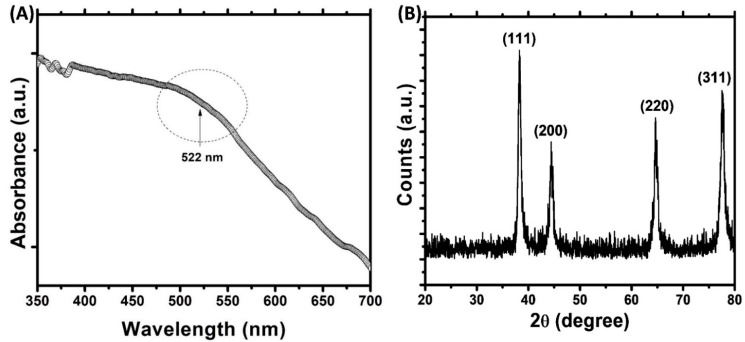
(**A**) UV-visible absorption spectrum of gold nanoparticles. Arbitrary unit (a. u.) (**B**) X-ray diffraction (XRD) pattern of gold nanoparticles.

In this study, a green route for the synthesis of gold nanoparticles was adopted. The phytocompounds present in the aqueous extract of *Trachyspermum ammi* seeds acted as reducing agent and capping agent for the synthesis. The microwave irradiation was used to accelerate the reaction process and to obtain the controlled size of AuNPs [27]. Initially, the color of HAuCl_4_ solution was light yellow that changed to ruby red after microwave irradiation. This change in color is due to the reduction of Au^3+^ and formation of gold nanoparticles [39]. The UV-vis spectrum of gold nanoparticles is presented in Figure 1A. The aqueous extract of *Trachyspermum ammi* seeds do not exhibit any prominent absorption band in the range of 400–700 nm [40]. The microwave irradiation of mixture of *T. ammi* extract and HAuCl_4_ caused the change of reaction mixture to ruby red indicating the formation of gold nanoparticles. The synthesis of gold nanoparticles is also evident from the UV-vis absorption spectrum. A broad band was recorded in 550–600 nm range which is indicative of the polydisperse nature of gold nanoparticles [41]. This absorption band is attributed to the SPR of the gold nanoparticles. Moreover, the finding is agreement with an earlier report where gold nanoparticles synthesized using olibanum gum exhibited SPR near 530 nm [27,42]. The compounds present in the extract of *T. ammi* acted as reducing and capping agents and are responsible for the reduction of Au^+3^ and synthesis of gold nanoparticles.

The FTIR spectrum of aqueous seed extract of *T. ammi* has been reported earlier that exhibited peaks at 2927, 1607, 1405, 1076, 617 cm^−1^ [40]. These peaks are due to the presence of soluble organic components of the extract which may be responsible for the synthesis of gold nanoparticles. Moreover, it is envisaged that presence of polyphenols such as thymol (major phytoconstituent of *T. ammi*) were responsible for bioreduction and capping process.

The presence of crystalline gold nanoparticles was confirmed by XRD analysis. The XRD pattern of gold nanoparticles is shown in Figure 1B. The presence of four diffraction peaks at 38.28, 44.42, 64.66, and 77.50° were observed. These peaks correspond to (111), (200), (220), and (311) sets of lattice planes that are indexed to face-centered cubic (fcc) crystal structure (JCPDS No. 04–0784). The absence of other diffraction peaks indicates that AuNPs were of pure crystalline. The nanoparticle size was calculated using Debye–Scherrer’s Equation (1) [33]:

(1) $$D_{p}=\frac{0.9\;\lambda }{\beta cos\theta }$$

*Dp* is crystalline size; *λ* is wavelength of CuKα (1.54060 Å); *θ* is Bragg angle; *β* is full width at half maxima (FWHM). The average crystalline size of gold nanoparticles was found to be 16.63 nm.

**Figure 2:**
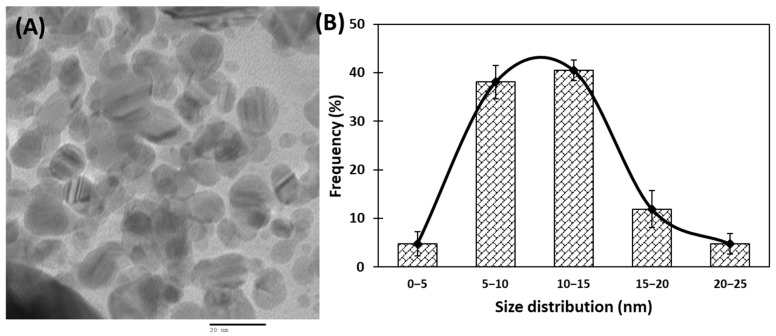
(**A**) Transmission electron microscopic (TEM) image of gold nanoparticles at 200,000× and 200 kV. (**B**) Frequency of gold nanoparticles size distribution.

**Figure 3:**
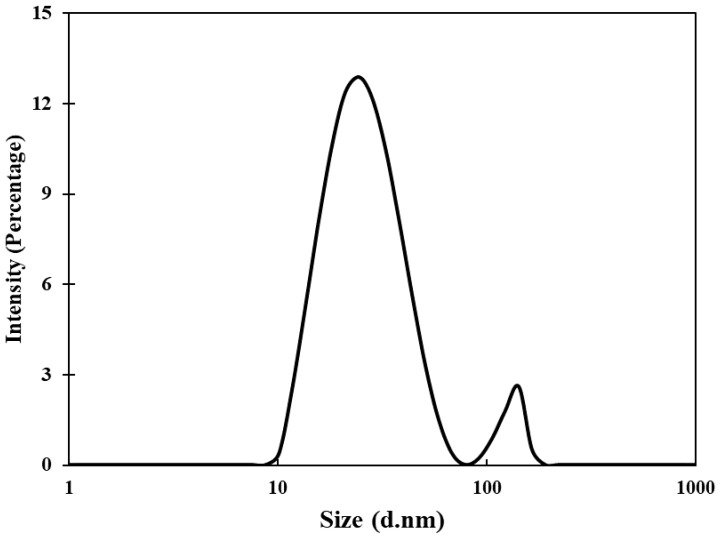
Hydrodynamic diameter of gold nanoparticles using dynamic light scattering (DLS) measurements.

Transmission electron microscopic (TEM) analyses of gold nanoparticles were performed to obtain the shape and size distribution of the particles. Transmission electron micrograph of gold nanoparticles is shown in Figure 2A. As evident from the figure, most nanoparticles were observed as spherical and spheroidal with few anisotropies. The size of particles ranged from 3 to 23 nm (Figure 2B). The average particle size using TEM analysis was found to be 11.11 nm. The size of green synthesized nanoparticles as well as their size distribution depends on relative nucleation rate and the extent of agglomeration [43]. The average range of the gold nanoparticles was similar to an earlier report in which gold nanoparticles synthesized using aqueous extract of *Garcinia mangostana* fruit were found to be 16 nm using XRD data [44]. Dynamic light scattering (DLS) measurements were carried out to further validate the size of nanoparticles. The average diameter of gold nanoparticles using DLS were found to be 24.4 nm (Figure 3).

### 3.2. Minimum Inhibitory Concentration (MIC)

Lowest concentration at which no visible growth is observed is termed as the MIC. In the present investigation, two food associated bacteria (*S. marcescens* and *Listeria monocytogenes*) were evaluated using the AuNPs fabricated from the seed extract of *T. ammi* (TA-AuNPs). Synthesized TA-AuNPs demonstrated MICs of 16 and 32 µg/mL against *S. marcescens* and *Listeria monocytogenes*, respectively. Higher MIC value exhibited by Gram positive *L. monocytogenes* in comparison to the *S. marcescens* (Gram negative) could be attributed to the difference in the structure and constitution of the cell wall. The cell wall of the Gram-positive bacteria is made of a thick layer of peptidoglycan with covalently attached teichuronic and teichoic acid making them less susceptible to the action of NPs [42,45]. Concentrations below MIC i.e., sub-MICs were considered for biofilm and virulence assays.

### 3.3. Biofilm Inhibition Studies

**Figure 4:**
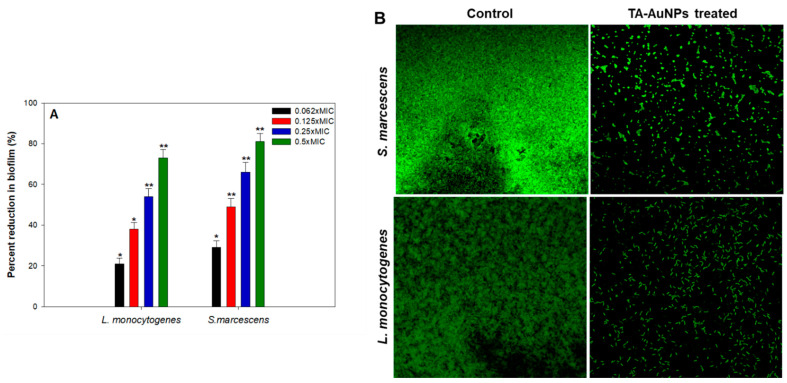
(**A**) Effect of sub-MICs of TA-AuNPs on biofilm formation. * denotes significance at *p ≤ 0.05* and ** denotes significance at *p ≤* 0.005. (**B**) Confocal laser scanning microscopic images of *L. monocytogenes* and *S. marcescens* biofilm in the absence and presence of 0.5× MICs of TA-AuNPs.

Aggregation of free-living planktonic bacteria through several physiological factors like motility, cell attachment, proliferation, differentiation, and accumulation of multilayered cell aggregates in a polymeric matrix leads to the formation of biofilm [32]. A plethora of research findings have demonstrated that the matrix acts as a barrier and protects the cells against the action of antibiotics, detergents, disinfectants, and host immune responses making them resistant [46]. Therefore, control of biofilm is of paramount importance. The results of the efficacy of TA-AuNPs in inhibiting the biofilm formation of *S. marcescens* and *Listeria monocytogenes* are depicted in Figure 4A. Concentration dependent biofilm inhibition by TA-AuNPs was observed against both foodborne pathogens. Maximum reduction of 81% and 73% in the biofilm formation was recorded at 0.5× MIC against *S. marcescens* and *Listeria monocytogenes*, respectively. Further, statistically significant (*p* ≤ 0.05) biofilm inhibitory activity of TA-AuNPs recorded at lower sub-MICs (0.062–0.25× MIC) against both the test pathogens is depicted in Figure 4A. Our observations corroborate well with findings on AuNPs fabricated using rhizome extract of *Rhodiola rosea* [47]. Synthesized AuNPs demonstrated significant biofilm inhibitory activity against *E. coli* and *P. aeruginosa* at sub-MICs. Further, in situ microscopic analysis was done using CLSM (Figure 4B) to validate the findings of the biofilm inhibition assay. Biofilms that were grown without TA-AuNPs treatment (control), exhibited highly multilayered aggregation of cells enclosed in an envelope of polymeric substances. On the contrary, treated cells of *S. marcescens* and *L. monocytogenes* were devoid of the characteristic biofilm architecture, scattered and disintegrated microcolonies were observed. Therefore, it is envisaged that the synthesized TA-AuNPs impedes biofilm formation in both *S. marcescens* and *L. monocytogenes* at sub-MICs.

### 3.4. Effect of Sub-MICs of TA-AuNPs Biofilm Related Virulence Traits

#### EPS

**Figure 5:**
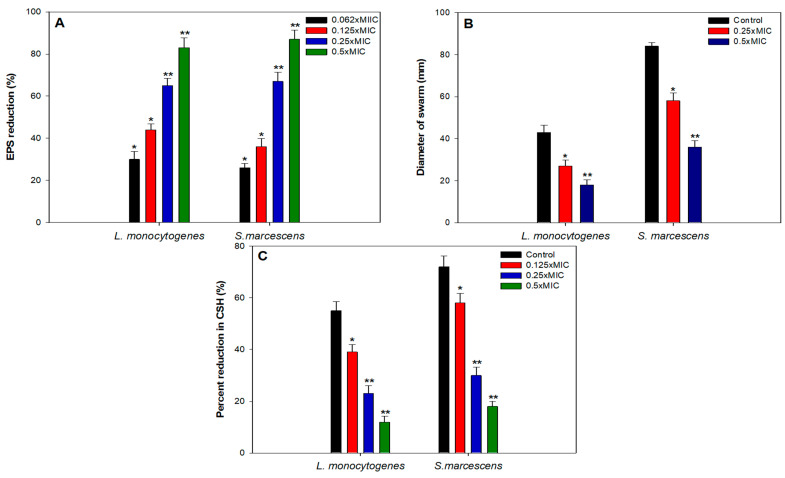
Effect of sub-MICs of TA-AuNPs on (**A**) EPS, (**B**) swarming motility, (**C**) cell surface hydrophobicity in *L. monocytogenes* and *S. marcescens*. Data are represented as mean values of triplicate readings and bar is standard deviation. * denotes significance at *p ≤* 0.05, and ** denotes significance at *p ≤* 0.005.

Exopolysaccharides (EPS) are an important constituent of the bacterial biofilm as they have a vital role in the maintenance of the 3D biofilm architecture and providing resistance against the action of antimicrobials and immune responses [48]. Several studies have highlighted that interference with EPS production leads to disturbed biofilm architecture and eventually reduces the development of resistance against antimicrobials [49,50,51]. In light of this, TA-AuNPs were evaluated for their ability to inhibit EPS production in *S. marcescens* as well as *L. monocytogenes*. EPS was reduced significantly in both the test pathogens in the presence of respective sub-MICs (Figure 5A). There was 83% and 87% reduction in the presence of 0.5× MICs of TA-AuNPs, respectively. In *S. marcescens*, 26%, 36%, and 67% impaired EPS production was recorded at 0.06× MIC, 0.125× MIC, and 0.25× MIC, respectively. Similar concentration dependent effect of TA-AuNPs was observed in *L. monocytogenes*, at concentrations ranging from 0.06× MIC to 0.25× MIC, 30–65% decrease in EPS was recorded. Our findings stand on par with the results reported with iron oxide nanoparticles, wherein, the nanoparticles reduced EPS production in *S. marcescens, E. coli, P. aeruginosa,* and *L. monocytogenes* by 15–80% at concentrations ranging from 2 to 16 µg/mL [52].

### 3.5. Swarming Motility

Initial attachment of cells to biotic or abiotic surfaces is crucial to biofilm development. Bacteria utilize flagellar driven swarming motility to adhere initially to the substratum. In both *L. monocytogenes* and *S. marcescens*, flagellar-driven motility is known to play a pivotal role in the initial attachment and subsequent biofilm formation [32,53,54,55]. Any intervention in the motility of the bacteria is bound to impact the attachment process negatively and eventually lead to impaired biofilm formation. In this regard, sub-MICs (0.06–0.5× MIC) of TA-AuNPs were examined for their efficacy in reducing swarming motility. Figure 5B depicts the concentration dependent suppression of motility in the *L. monocytogenes* and *S. marcescens*. Highest decrease of 58% and 64% in the swarming behavior of *L. monocytogenes* and *S. marcescens* respectively, was recorded as compared to their untreated controls. Our results find support from the observations made with sub-MICs of AgNPs, ZnO nanoparticles and ZnO-xanthan gum nanocomposite, wherein a significant drop in the swarming motility behavior was recorded in test pathogens [49,56,57,58].

### 3.6. Cell Surface Hydrophobicity (CSH)

Hydrophobicity index is the measure of the charge carried by the cell surface, it is one of the key elements that facilitates the process of accumulation, aggregation, and attachment of cells. In view of this, it is envisaged that by reducing the hydrophobicity index of the cells, the accumulation and aggregation of the cells will be reduced and consequently impair biofilm formation [55]. Thus, we assessed the effect of sub-MICs of TA-AuNPs on the CSH of both *S. marcescens* and *L. monocytogenes* using MATH assay. Obtained data exhibited concentration dependent significant reduction in CSH as shown in Figure 5C. In case of *S. marcescens*, untreated control showed 72% CSH, while, treatment with 2, 4, and 8 µg/mL resulted in 58%, 30%, and 18% CSH. Similarly, *L. monocytogenes* also demonstrated decreased CSH ranging from 39% to 12% upon treatment with 0.125–0.5× MIC of TA-AuNPs. This drop in the hydrophobicity of test pathogens could plausibly be one of the reasons for the biofilm impediment observed in the earlier assays. Data obtained agrees with the findings on zinc oxide-xanthan gum nanocomposite wherein, only 21% and 16% CSH was recorded in *C. violaceum* and *S. marcescens* upon treatment with 0.5× MICs [58]. In another study conducted on *P. aeruginosa* biofilm using Cu nanoparticles, reduced biofilm formation in *P. aeruginosa* was attributed to the decreased CSH [59].

### 3.7. Effect on Preformed Biofilms

**Figure 6:**
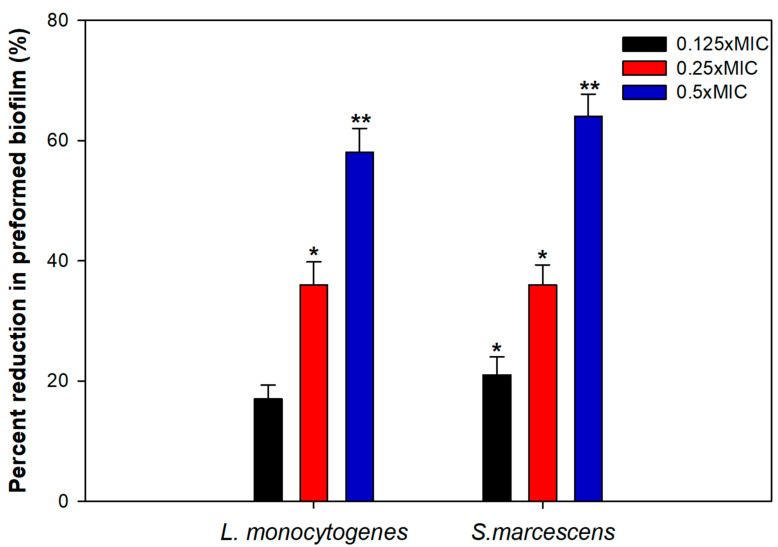
Disruption of preformed biofilm by sub-MICs of TA-AuNPs. * denotes significance at *p ≤* 0.05, ** denotes significance at *p ≤* 0.005.

Bacterial cells residing in the biofilm mode can tolerate multiple stress conditions and are almost 1000 times more resistant to bactericidal agents as well [60]. Thus, the disruption of preformed mature biofilm is rather difficult and hence, we evaluated the ability of TA-AuNPs to obliterate mature biofilms of *S. marcescens* and *L. monocytogenes*. The results clearly demonstrated a significant level of biofilm disruption in the test pathogens at tested sub-MICs of TA-AuNPs (Figure 6). In *S. marcescens*, preformed biofilm was reduced by 21%, 36%, and 64% upon treatment with 2, 4, and 8 µg/mL concentration of TA-AuNPs. Preformed biofilms of *L. monocytogenes* witnessed a drop of 17–58% over untreated control at concentrations ranging from 4 to 16 µg/mL (0.125–0.5× MIC). Mature biofilm possesses a robust EPS matrix that resists the action of antimicrobials and host immune response. Our results indicate that the synthesized AuNPs interfere with the EPS matrix, disintegrate the architecture of the biofilm, and make the cells susceptible to antibiotics. Nanocomposite comprising AuNPs and reduced graphene oxide (Au-RGO) demonstrated similar concentration dependent obliteration of preformed mature biofilm of *L. monocytogenes*, MRSA, *S. marcescens*, *E. coli,* and *P. aeruginosa* at concentration ranging from 0.25 to 0.5× MICs [61]. Our results are in accordance with those reported with the Au-RGO.

### 3.8. Mechanism of Biofilm Inhibition

Effect of highest tested sub-MIC (0.5× MIC) of TA-AuNPs on the growth of *S. marcescens* and *L. monocytogenes* was assessed and no significant reduction in the viability of the bacteria was observed (data not shown). Thus, the reduction in biofilm upon treatment with sub-MICs of TA-AuNPs is plausibly not due to the killing of the bacterial cells.

**Figure 7:**
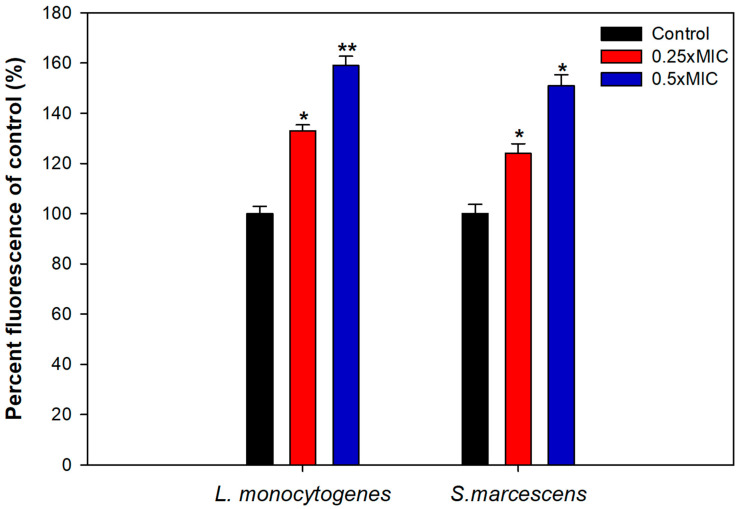
Intracellular ROS generation induced in test bacterial pathogens treated with sub-MICs of TA-AuNPs. * denotes significance at *p ≤* 0.05, ** denotes significance at *p ≤* 0.005.

To uncover the plausible mechanism of biofilm inhibition, intracellular ROS generation in TA-AuNPs treated cells was evaluated. Considerably elevated ROS levels were observed in cells of *S. marcescens* and *L. monocytogenes* treated with 0.25–0.5× MICs of the AuNPs (Figure 7). Intracellular ROS production increased by 59% and 51% in *L. monocytogenes* and *S. marcescens*, respectively, treated with 0.5× MICs of AuNPs. Increased ROS production is one of the most important mechanisms through which AuNPs disrupt the normal functioning of bacterial cells. Thus, it is envisaged that the interaction between bacterial cells and synthesized AuNPs induces higher ROS production due to metabolic imbalance in the cells. Increased ROS levels overpower the antioxidant defense system of the cells leading to oxidative stress, initiating lipid peroxidation, cell membrane damage, and finally cell death [62]. Our study elucidates that the TA-AuNPs induces an upsurge in the production of intracellular ROS in *L. monocytogenes* and *S. marcescens* that exceeds the capacity of antioxidant system of the cells and leads to cell death.

## 4. TA-AuNPs Inhibits Growth and Viability of HepG2 Cancer Cells

**Figure 8:**
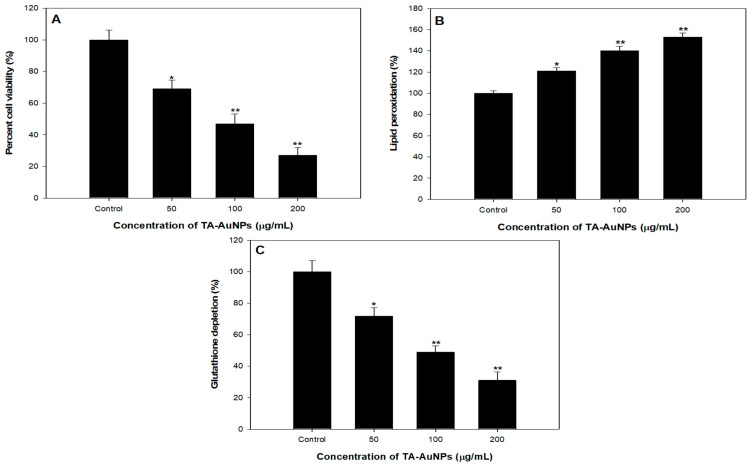
Anticancer studies against HepG2 cell lines. (**A**) Cytotoxicity assessment by MTT assay. (**B**) Effect on lipid peroxidation. (**C**) Percent change in glutathione levels. * *p* < 0.05, ** *p* < 0.005.

To scrutinize the antiproliferative prospective of TA-AuNPs, we executed 3-(4, 5-dimethythiazol-2-yl)-2, 5-diphenyl tetrazolium bromide (MTT) assay against HepG2 cancer cells. It was observed that TA-AuNPs administration (0–100 μM for 24 and 48 h) to HepG2 cells results in inhibition of cell growth in a concentration and time-dependent approach. Time course scrutiny revealed HepG2 cells respond to TA-AuNPs treatment within 48 h. The IC_50_ value of TA-AuNPs treated HepG2 was 92.453 µg/mL. The data suggested that TA-AuNPs treatment showed a significant potential in inhibiting proliferation of HepG2 cells (Figure 8A). Cellular propagation consequential in tumor pattern might occur because of the alteration in cell cycle regulation. A crucial primary origin of cancer succession is recognized by speedy and candid proliferation results to series and expansion of tissue accrual [63,64]. Results of MTT assay specified that TA-AuNPs is certain in its activity and proficient against cell cancer cell lines from different derivation. Cell growth in HepG2 cells is transformed by TA-AuNPs. Administration of TA-AuNPs resulted in the arrest of cellular proliferation in a concentration-dependent manner; magnification in the hammering of cell viability was experiential with amplification in the concentration of dose.

## 5. Effect of TA-AuNPs Treatment on Lipid Peroxidation in HepG2 Cancer Cells

Lipid peroxidation induced by reactive oxygen species (ROS) generation plays a crucial part in cell death including autophagy and apoptosis. This essential and preserved mechanism is based on an excessive production of ROS, which leads to impairment of bio membranes, encourages lipid peroxidation chain reactions, and consequently persuades cell death. We observed a dose dependent effect of TA-AuNPs on lipid peroxidation in HepG2 cancer cells. A noticeable increase in Lipid peroxidation with increase in concentration of TA-AuNPs (100 and 200 µg/mL) treatment was seen in HepG2 cancer cell lines (Figure 8B).

## 6. TA-AuNPs Treatment Leads to Depletion of Intracellular Glutathione (GSH) of HepG2 Cancer Cells

GSH redox is a vital element in most of the biological processes like regulation of cell-proliferation and apoptosis, control of various signal transduction pathways, initiation of genes at transcription level. Our results revealed that administration of TA-AuNPs significantly depleted the GSH levels in HepG2 cancer cells (Figure 8C), which signifies that TA-AuNPs may acts as potent anticancer therapy. At 200 µg/mL, the statistically significant depletion of 75% in GSH levels was seen while at 100 µg/mL, 56% reduction in GSH levels was seen after treatment of TA-AuNPs in HepG2 cancer cells (Figure 8C). Redox status (one major component being GSH) is a major determinant of metastatic aggressiveness and sensitivity to chemotherapeutic agents. GSH deficit, or a reduction in the GSH/glutathione disulphide (GSSG) ratio, results in an amplified susceptibility to oxidative stress associated with the growth of cancer, raised GSH intensities increase the antioxidant capability and the resistance to oxidative stress as pragmatic in various types of cancer cells [65]. GSH is a documented marker of oxidative stress and leading scavenger of ROS. Our results support previous studies by decreasing the intracellular GSH in HepG2 cancer cell lines after treatment of TA-AuNPs. Therefore, posing cells become more prone to ROS generation, hence induce apoptosis. The overall results provide preliminary confirmation of the view that cellular GSH expression in cancer cells may be a target for therapeutic operations [66]. On the basis of our results, we propose that TA-AuNPs may act as an anticancer therapeutic agent.

## 7. Conclusions

The research work embodies a rapid microwave-assisted green synthesis method of gold nanoparticles using the seed extract of *T. ammi* as capping and reducing agent. Our data throws light on the biofilm inhibitory potential of the synthesized NPs at SUB-MICs against pathogens, *L. monocytogenes* and *S. marcescens*. TA-AuNPs were found effective in thwarting virulence factors like EPS, swarming motility, and CSH that facilitates the formation and maintenance of biofilms. Further, the NPs not only inhibited biofilm formation but also disrupted robust preformed biofilms of the test pathogens. The study describes the enhanced ROS production as the plausible mechanism of antibiofilm action. Further, these NPs were found to be effective anticancer agents against HepG2 cancer cell lines. Thus, we can conclude that the synthesis route is environmentally friendly and the synthesized AuNPs possess significant antibiofilm and anticancer potential that can be exploited in various biomedical applications.

## Data Availability

The data presented in this study are available on request from the corresponding author.
